# Supplementary material for: Molecular characteristic of activin receptor IIB and its functions in growth and nutrient regulation in Eriocheir sinensis
Source: PeerJ. 2020 Sep 1;8:e9673. doi: 10.7717/peerj.9673 (PMC7473049; doi:10.7717/peerj.9673)
Supplement: Supplemental Information 1 — The start codon (ATG) and the stop codon (TAA) were indicated with bold letters in box. The mRNA instability motif (ATTTA) was showed by bold letters. The signal peptide sequence was double underlined. The activin receptor domain was shaded with light gray. The transmembrane region was indicated in box. Serine/Threonine protein kinases domain was showed with a bold underline. [file peerj-08-9673-s001.pdf]

1	AATACCCCATGGAAATTTTAAACAGCCAAA
31	TATACACCAGTAAAACCCATGTAATCACCCTTAAAAACCCGCAGCGGAGGTTTTTAGAGGACTCCCCGCCAGTGGATTATGTCT
115	CCTGAAAATACTCGCCATATTGGATTTCGCTAACAAATCGGGCCTGAAATACTGCTGCTGGGCGAGGCATTGCTTGACGCTGCA
199	CCCTGCGGGCCCCGAGGCGAGTGGCCCAGCGCACTGTGATCGGTGGGGGGGCACGAGCAGGTGATGGTGTGCTGCAGCTGCTGC
283	AACAAGTGTGACAGTGCTCAAAATTACCCTTTCCATGAAGAAGGATACAGTGAAAAATCTAGAACCCATGTGCTTTGTTTTACA
367	CAGTGAAGTTCCTCTTAAAATCTGTTGCTAAAGTGAAGGAAAAACAGCTTGATGAGAGCCCGAGAGGGTTGCCATCGATGCAGG
451	ATATGGACATGACACGGGTCTGTCCATGAGGACAGATGTGACATTTGGAGGAGCTGAGGTGCCACACATGCAAGACCTGACTGG
535	ATCCTGGCTGAGAGGGATCACCCAAGGACAAGTGAGGGGTGGCTGAAGCTGAGGAGACCCAAGGCAAGGTTGGCCAGCTGTGTG
619	**ATG**GCTGGCAGCAGAAGAGTCTTGTATCCTCCATCAACCATGATGATCCTGCTAACCCTGGCAGCCTTCCCTGCCATCTCGGCC
1	M A G S R R V L Y P P S T M M I L L T L A A F P A I S A
703	CTAATACCTGAAGGGATGGCACAGCCTTCGGAAACCCCCCGGCCCCAGGTGACGCACTGTGAGCTATACAACAGCACACAGTGT
29	L I P E G M A Q P S E T P R P Q V T H C E L Y N S T Q C
787	ACTGGGGACAACTCTCTCTCGCCTGTGTGTAAGAAGGAAAGCCATCCGTGTGAGGACCTAGAGAAGGCCAACCAGTGCTTTGTG
57	T G D N S L S P V C K K E S H P C E D L E K A N Q C F V
871	GTGTGGAGTAACATGTCTGGCACGCCAGAAGTAATCTACAAGGGATGTTTATGGAATTATAAAACTTGCAAAGATGAGTGCATC
85	V W S N M S G T P E V I Y K G C L W N Y K T C K D E C I
955	AGCACTGAGCCAGTGAACAGTCTTACAGAGCAGAAGCCTCACCTGTTTTGTTGCTGCAACAACAACAATTGTAATCAGAACTTC
113	S T E P V N S L T E Q K P H L F C C C N N N N C N Q N F
1039	TCGTGGCAGCCTAAGGTTGAGGTCCCCAGCACCACCCCAGAGGGGCCTTTAAGAAACATGATCCCCGCAGCTACTGGGTTAATG
141	S W Q P K V E V P S T T P E G P L R N M I P A A T G L M
1123	GCCCAGGGTAACAGGGCTTACACTGGAAAAAGGGATAAAAACCTTATAAACATCCTGCCACCCCAGGAACAGGACATGGTTGTA
169	A Q G N R A Y T G K R D K N L I N I L P P Q E Q D M V V
1207	CAGACGGTGGCTTGGACCCTTGGGACCCTCATCCTGTTGGTGGTTACGGTGACAGTTCTCTTCTACCTCTATAGGAGACAGAAA
197	Q T V A W T L G T L I L L V V T V T V L F Y L Y R R Q K
1291	ATGGCCAACTTTATGGCAATACCTCGAGTGGAGTCCACAGCCCTTGTTCCTCCCTCTCCACCGATGGGCTTGCGACCCATACAG
225	M A N F M A I P R V E S T A L V P P S P P M G L R P I Q
1375	CTGAGGGAGATCAAAGCCAGAGGCCGGTTTGGTGCTGTGTGGAAGGCCAACCTTCACAATGACGTTATTGCTGTCAAGATCTTC
253	L R E I K A R G R F G A V W K A N L H N D V I A V K I F
1459	CCAGTCCAGGATAAACAGTCGTGGCTGGTGGAGACGGAGGTGTACTCCCTCCCTCAGCTGTCCCATGAGAATATCCTACACTAC
281	P V Q D K Q S W L V E T E V Y S L P Q L S H E N I L H Y
1543	ATTGGGGCAGAGAAGCGTGGCGATAGCCTTCAGGCTGAGTTTTGGCTTATTACAGCCTACCATGAGAGAGGCTCCTTGTGTGAC
309	I G A E K R G D S L Q A E F W L I T A Y H E R G S L C D
1627	TTTCTAAAGGCCAACCTCGTGACATGGGATGAACTGTGCAAGATTGGCGAGTCAATGGCCCGGGGGTTAATGTACATGCATGAG
337	F L K A N L V T W D E L C K I G E S M A R G L M Y M H E
1711	GAGCAACCGGCTTCCAAGTGTGAGGCCCTCAAGCCTGCCATTGCCCACCGAGACTTCAAAAGCAAAAATGTGTTGCTGAAGAAT
365	E Q P A S K C E A L K P A I A H R D F K S K N V L L K N
1795	GACCTGACTGCCTGCATTGCTGACTTCGGCCTTGCTTTGACCTTCCACCCTGGACAGTCAACTGGTGACACTCATGGACAGGTG
393	D L T A C I A D F G L A L T F H P G Q S T G D T H G Q V
1879	GGCACAAGGAGGTACATGGCCCCTGAAGTCTTGGAAGGGGCCATCAATTTCCAGCGTGATGCCTTCTTACGCATTGACATGTAT
421	G T R R Y M A P E V L E G A I N F Q R D A F L R I D M Y
1963	GCCTGTGGCCTTGTGCTGTGGGAGCTGCTGTCCAGATGTTCAAGCCCAGATGGACCCATACCTGAGTACCACTTGCCATTTGAG
449	A C G L V L W E L L S R C S S P D G P I P E Y H L P F E
2047	GAGGAAGTTGGCCAGCATCCAACATTGGACGACATGCAGGAGTGTGTCGTCACCCAAAAGGCTCGACCTGTCATCCACGACCAT
477	E E V G Q H P T L D D M Q E C V V T Q K A R P V I H D H
2131	TGGCGGAAGAATGCTGCCATGATGGGATTGATAGACACCATGGAGGAGTGCTGGGACCATGATGCAGAGGCCCGTCTCTCAGCT
505	W R K N A A M M G L I D T M E E C W D H D A E A R L S A
2215	TCATGTGTGGTGGAGAGGCTGGCCAGCTTCTCAAGGAACCCCCAGTTTTCCCCTACCTCAAATCCACAGAAGGAGTCGAGTATA
533	S C V V E R L A S F S R N P Q F S P T S N P Q K E S S I
2299	**TAA**GGCCAAGGTGTGGTGGCCACGTACCTGGACAAGTACCTGTGCTGTAACCGCCTATGAGGGTCCTGGTGCGGGGGAGAGCCC

561 *

2383 AGCCACCATGTCCCGCAGGCACCAGCTGTGTTGGGGGCCTCGTGTGTCGGCCTCAGTAACCCTCCCCCCTCCCTTCTCCAATCA

2467 GCTGCACACCACAGCCAGACACTTGGTCATATTCACAGTTACCCGGTGACACCAGTTTTGTCTGGTCAAAGAGGGTTGGGTTCA

2551 GGGAATTTCTCTTCACATTTGAAAAAAAAAAGTACTTTTACTCTCAGCATCTAAATTGGCACTGATGGGTGAGAGTCTCCGTTA

2635 GTTAGATAGATACGTCTTCCTGTAATTAATTAGTGCAATAGTTTGATATCCCTGGCGGTGCTTCAAATGCATTTTTATGGACTG

2719 ATGCAAAAATCATACTTAGCGGGAAGTAAATTTTGGGATCCTATAGTCCAACCATAATTGATCTGGCTTGTGTGGTTGGGAGAG

2803 GGGGGAGGCTTAAAGTGTGGTGCCAAAGGGGTCAACAGGGAAGTTCTGGGACTGTTAGATAGAGCCACCCAAGTGAAGGTAGCT

2887 TGTGGGAATTCACTCCATGGCCTCATGACTCCCCATAGGTTGAGATGCTCCATAACACTACCCTCTCCAGCATTATTGTTGGGA

2971 TCATGTTTCCTTGTGTTGGGCTTGAAAGGGTTAACAAT**ATTTA**GCTGTTATTATTACTGTCCAAAAAAGAAATTGATGTTCTTT

3055 GAAATACTTACACTTATTGGTATCGGCTGTTT**ATTTA**TGTTACGAACATGCAAGAAGGAAACCCTGTAC**ATTTA**GTGCAGACTA

3139 TAGCATGCGCAGCTGCTAACATAGAAGAATGAAAGAGGATCTCATGGTGCGTGTTGCCATAATGGAAGAAGCACCAAGAGGTAA

3223 AATTATTTTCGTAAAGTATCTAAATAAAATTTCAAGAGGAATGTTTTCACGTGCCACCCACACAGGGACCGTCTTGGGCATTCC

3307 TACCTCTGAATCCTCACTGTAAGTCTCCTGATGTGAGTTGAGGAGCTGTGGTGCTGCTTCCATCTTGTAGGACGGCTGTGGAAA

3391 GTGAATTCTGAATCTTCATCACCATCCGATTGTTTGGTGTGTACGATGGAGCTGGAGATCGTGCATGGAAAGTGGTGTTCACAG

3475 CTGTCAGTGCATTTCCTGTCAATGAGAAACATGGTGTCT**ATTTA**ACCATCAACAGACAATTGCATCCAGCCATTCAGTTTTGCA

3559 GCTCAGTGCAGCTTCAAGCTCAGTAACTCAGGCAAAAGGAAAACTATGGTTGTGCCTCCCTGGCAAGTGTCTAATGGCGTATGG

3643 GGAAGAGTGGCTGTGGGTTCGTCTGTCCTAAACTGAAATTTCCCATCTGTGTTGAAAACTGGATGGATTGCAGTGTTCTAATCA

3727 GTGAAGCTTTTGTTTTTAATTTTTGCTTAGAAAGACTCTATAGTGTGAATGATTTCTACATAAAAAGGAAACATAGTATTTCAG

3811 CCCACTATGTTCATTATCTGTGTGTCGTCAGTGCCAGCAGAGCTCCGACACTGTCTGCTGGGCTAGTAGGACACAAGACGAGCC

3895 CAGCCATTGTGAGGCCAGTGGAGGGCCACAATCGGGCTGCCCCTTTACTGTCAGCTCAGGACAAACTTCAGTGACCACCCTGTT

3979 ACCACTTTGCCCCCACACACGTACATACATACCCTCATCTTGTATATATTTGCGCCTTGTTTGTAGCATTACACAAGTTGTCGC

4063 CAACAAAATGAAACTAGTGATTATTTTAAATGTGGTTTATTTGTGAAACCAATGGTGCTTTTTTTCTTCTATTTCCAAGATGAA

4147 TGTTAATTGTTAAACACTGAATCAAGCCAGCATGAGTGTGGAAACAAAGAGGCACATTGCCCACTCAGCATTGTAAATACCTCA

4231 TTATTGTACACAGCATTATGACTTGTGACATCCAGACCTCACCATGACAGATCCTGAATGCTTATAAGCAGAATTCTATTACTT

4315 CTTGATTGTGTATAGTAAGTTCTCTCTTGTTTGGGAACTATAGTTGTTGGAAAGCCATAGAAAGGTCATTTTATTGATCCAGAA

4399 AACTTAAAAGAGGAAACTAAAGTAAACTCTGGTCTCTGCA**ATTTA**ATTCTTGTCATTGTAGTTAAGCCATTAAGAGGCTATGAG

4483 AGAGAAAGAGAGAGAGAGAGACTGTAAAATGAGAAAATAGATAAAAGCAAAGAGAAAATTGAACTTTGCAAAACTTTGACCTTG

4567 GGAGACTTGAGTTCATCTGCTGTGAGGCCTTCTGAGGCGAGGACGAGGCAGTGTGACTGTCAAACGAGGACTGGAGGCGTCATG

4651 TGATAGATTGGATACGTACAGGGACTTTTGGGCACAGGTACCGCCTGTGCCTTGGGGAACCACTTGTCACTGTTACAAGACTGT

4735 GTATGGGGGGAAATTTGCTATTTTCTTGTACTTCCTTGAATTA**ATTTA**ATTTTATTTTATGTTCATTACTTGTTAGGACACCCC

4819 CTTTATCCTTGTTGTTCCTTGCAGGCATGAATGTAATGTCTCATATATCTGTATCCCATGTAGTAGTATAAAGTAATCGATATT

4903 GAAAAAAAAAAAAA
