# Supplementary material for: Molecular characteristic of activin receptor IIB and its functions in growth and nutrient regulation in Eriocheir sinensis
Source: PeerJ. 2020 Sep 1;8:e9673. doi: 10.7717/peerj.9673 (PMC7473049; doi:10.7717/peerj.9673)
Supplement: Supplemental Information 2 — RACE: rapid-amplification of cDNA ends; qRT-PCR: quantitative real-time polymerase chain reaction. [file peerj-08-9673-s002.docx]

**Table S1**

**The information of primers used in this article.**

RACE: rapid-amplification of cDNA ends; qRT-PCR: quantitative real-time polymerase chain reaction.

| Primer name | Sequence (5′-3′) | Usage | Fragment size | GenBank  accession No. |
| --- | --- | --- | --- | --- |
| ActRIIB-F | CAACAAGTGTGACAGTGCTCAA | PCR | 2272 bp | MN832896 |
| ActRIIB-R | CCCTGAACCCAACCCTCTTT | PCR |  |  |
| ActRIIB-5′outer | CAACCTTGCCTTGGGTCTCCTC | RACE | —— | MN832896 |
| ActRIIB-5′inner | CACCTCAGCTCCTCCAAATGTCA | RACE |  |  |
| ActRIIB-3′outer | GAGGACGAGGCAGTGTGACT | RACE | —— | MN832896 |
| ActRIIB-3′inner | GGGAACCACTTGTCACTGTTAC | RACE |  |  |
| 5′-UPM | AAGCAGTGGTATCAACGCAGAGT | RACE | —— | MN832896 |
| 3′-UPM | CTGATCTAGAGGTACCGGATCC | RACE |  |  |
| T7-dsRNA-ActRIIB-F | gatcactaatacgactcactatagggGGCCAACCTTCACAATGAC | dsRNA | 777 bp | MN832896 |
| dsRNA-ActRIIB-R | GGTGTCTATCAATCCCATCA | dsRNA |  |  |
| dsRNA-ActRIIB-F | GGCCAACCTTCACAATGAC | dsRNA | 777 bp | MN832896 |
| T7-dsRNA-ActRIIB-R | gatcactaatacgactcactatagggGGTGTCTATCAATCCCATCA | dsRNA |  |  |
| ActRIIB-RTF | GCCTCATGACTCCCCATAGGTTG | qRT-PCR | 244 bp | MN832896 |
| ActRIIB-RTR | TGCGCATGCTATAGTCTGCACT | qRT-PCR |  |  |
| β-actin-RTF | TCATCACCATCGGCAATGA | qRT-PCR | 100 bp | HM053699.1 |
| β-actin-RTR | TTGTAAGTGGTCTCGTGGATG | qRT-PCR |  |  |
| S27-RTF | GGTCGATGACAATGGCAAGA | qRT-PCR | 105 bp | HM177456.1 |
| S27-RTR | CCACAGTACTGGCGGTCAAA | qRT-PCR |  |  |
| UBE-RTF | TTGCGTTCACAACTCGTATCTACC | qRT-PCR | 137 bp | HQ436509.1 |
| UBE-RTR | GTCCGTGAGGAGGGAACAGA | qRT-PCR |  |  |
| ActRI-RTF | TGGCTTCTACGGCTCGGACAT | qRT-PCR | 111 bp | asmbl_20041 |
| ActRI-RTR | AGGGTGTGGGTGGTGAGGTA | qRT-PCR |  | (**Data S2**) |
| SMAD3-RTF | GGCCGTCTACCAACTCACTA | qRT-PCR | 126 bp | KY858966.1 |
| SMAD3-RTR | GGGCCATTCAAGTGTAGCTC | qRT-PCR |  |  |
| SMAD4-RTF | AGTGCCACCGTCAGATGCAG | qRT-PCR | 85 bp | TR74101_c0_g1_i1 |
| SMAD4-RTR | GAGATCTGACCCGCCACAGC | qRT-PCR |  | (**Data S2**) |
| FoxO-RTF | GAAGAAGATGGAGGCGTTAAGG | qRT-PCR | 80 bp | Esin016076.m1 |
| FoxO-RTR | ACAGGTCCAGGTTCTCATTCAT | qRT-PCR |  | (**Data S2**) |
| mTOR-RTF | TGAGCATGCGCTGGAACACA | qRT-PCR | 93 bp | asmbl_65644 |
| mTOR-RTR | GTGCGCCGATCAAACCACAC | qRT-PCR |  | (**Data S2**) |
| CPT1β-RTF | GCCTCTGATGGATGACGAGA | qRT-PCR | 160 bp | MH037159.1 |
| CPT1β-RTR | TCCTCGCAGGTACACAAACT | qRT-PCR |  |  |
| FAS-RTF | CCAGGTGGCTCGGGCATTAG | qRT-PCR | 88 bp | KM042200.1 |
| FAS-RTR | AGCGGTGCCGAAGGAATAGC | qRT-PCR |  |  |
| FAE-RTF | ACGAACTTCCTCCCACTCCG | qRT-PCR | 87 bp | KR005628 |
| FAE-RTR | GGAGAGGCTTGCTTCACACTAA | qRT-PCR |  |  |
