# Supplementary material for: Molecular characteristic of activin receptor IIB and its functions in growth and nutrient regulation in Eriocheir sinensis
Source: PeerJ. 2020 Sep 1;8:e9673. doi: 10.7717/peerj.9673 (PMC7473049; doi:10.7717/peerj.9673)
Supplement: Supplemental Information 3 [file peerj-08-9673-s003.docx]

**Table S2****:**

**The GenBank accession number of species** **used in phylogenetic tree.**

| Species | GenBank  accession No. | Species | GenBank  accession No. |
| --- | --- | --- | --- |
| *Eriocheir sinensis* | MN832896 | *Oncorhynchus nerka* | XP_029516925.1 |
| *Portunus trituberculatus* | MPC26231.1 | *Nasonia vitripennis* | XP_001603863.1 |
| *Penaeus vannamei* | ROT74806.1 | *Apis cerana cerana* | PBC29173.1 |
| *Daphnia magna* | JAL80963.1 | *Drosophila persimilis* | XP_002013935.1 |
| *Xenopus laevis* | AAB00480.1 | *Zootermopsis nevadensis* | KDR23233.1 |
| *Xenopus tropicalis* | NP_001006871.1 | *Atta colombica* | KYM81630.1 |
| *Nanorana parkeri* | XP_018408985.1 | *Cimex lectularius* | XP_014240288.1 |
| *Microcaecilia unicolor* | XP_030054017.1 | *Gallus gallus* | ARX98262.1 |
| *Rhinatrema bivittatum* | XP_029444276.1 | *Columba livia* | XP_021156889.1 |
| *Chelonia mydas* | XP_007059493.1 | *Picoides pubescens* | XP_009896151.1 |
| *Chrysemys picta bellii* | XP_005297214.1 | *Melopsittacus undulatus* | XP_005150084.1 |
| *Terrapene carolina triunguis* | XP_024057745.1 | *Calypte anna* | XP_008498969.1 |
| *Gavialis gangeticus* | XP_019363647.1 | *Phaethon lepturus* | XP_010281947.1 |
| *Pelodiscus sinensis* | XP_006120616.1 | *Mus musculus* | NP_031423.1 |
| *Danio rerio* | AAH97042.1 | *Ovis aries* | XP_011954784.2 |
| *Ctenopharyngodon idella* | ACI23559.1 | *Bos taurus* | NP_776920.1 |
| *Astyanax mexicanus* | XP_022529595.1 | *Orcinus orca* | XP_004277948.1 |
| *Oncorhynchus kisutch* | XP_020350020.1 | *Pongo abelii* | XP_002813969.1 |
| *Salmo salar* | ABK54368.1 | *Homo sapiens* | BAA24180.2 |
